# Preclinical Application of Conditional Reprogramming Culture System for Laryngeal and Hypopharyngeal Carcinoma

**DOI:** 10.3389/fcell.2021.744969

**Published:** 2021-10-29

**Authors:** Yanbo Dong, Jian Wang, Wei Ji, Mengzhu Zheng, Peng Wang, Liangfa Liu, Shanhu Li

**Affiliations:** ^1^Department of Otolaryngology Head and Neck Surgery, Beijing Friendship Hospital, Capital Medical University, Beijing, China; ^2^Department of Cell Engineering, Beijing Institute of Biotechnology, Beijing, China

**Keywords:** conditional reprogramming, head and neck squamous cell carcinoma, *in vitro* model, drug sensitivity, personalized treatment

## Abstract

Management of laryngeal and hypopharyngeal squamous cell carcinoma (LHSCC) remains highly challenging due to highly variable therapeutic responses. By establishing an *in vitro* model for LHSCC based on conditional reprogramming (CR), a cell-culture technique, we aim to investigate its potential value on personalized cancer therapies. Herein, a panel of 28 human LHSCC CR cells were established from 50 tumor tissues using the CR method. They retained tumorigenic potential upon xenotransplantation and recapitulated molecular characteristics of LHSCC. Differential responses to anticancer drugs and radiotherapy were detected *in vitro*. CR cells could be transformed to xenograft and organoid, and they shared comparable drug responses. The clinical drug responses were consistent with *in vitro* drug responses. Collectively, the patient-derived CR cell model could promisingly be utilized in clinical decision-making and assisted in the selection of personalized therapies for LHSCC.

## Introduction

Malignant tumors arising from the epithelium of the larynx and hypopharynx are predominantly squamous cell carcinomas, constituting the most frequent malignancies of head and neck squamous cell carcinomas (HNSCC). The management of LHSCC remains very challenging due to highly variable treatment response to radiotherapy and chemoradiation therapy. Insensitive to radiotherapy or chemotherapy frequently occurs, one of the significant causes of tumor recurrence, and is associated with poor outcome. Thus, identifying a precise and optimal treatment strategy for individual patients is highly desirable.

Conditional reprogramming (CR) system is a platform to establish a long-term culture of primary epithelium cells derived from normal and tumor tissues (Liu et al., 2012, 2017). The reported advantages of CR include high success rate, exponential growth, genotype stability, and ease of manipulation. These characteristics enabled CR as an exceptional *in vitro* model compared with the others, such as patient-derived xenotransplantation (PDX) and organoids. Owing to these advantages, CR has been documented by two American National Cancer Institute programs: patient-derived cancer model repository (PDMR) and human cancer model initiatives (HCMI) (Palechor-Ceron et al., 2019). Potential applications of the CR system in clinical settings have been investigated in breast (Mahajan et al., 2017), lung (Gao et al., 2017), prostate (Saeed et al., 2017), bladder (Kettunen et al., 2019), gastric (Zhao et al., 2021), and liver (Su et al., 2019) cancers. Recently, Liu et al., have also established one primary cell line using CR system derived from tongue squamous cell carcinoma, a type of head and neck cancer (Palechor-Ceron et al., 2019). However, long-term culture of laryngeal and hypopharyngeal tissues using CR system has not been reported. Although the CR system is robust, the application of CR cell lines was lack of evidence since much focus was on the establishment methods. Moreover, the clinical relationship of CR has not been completely validated.

The present study established a primary culture system that enabled the rapid amplification of genetically stable LHSCC cells with a high success rate. As a versatile *in vitro* model, CR could be transformed into organoids and be used to produce CR-derived xenografts. Both systems share comparable drug responses. This study aims to provide a preliminary investigation into the relationship between *in vitro* CR cell responses and clinical responses, which may contribute to its potential clinical implications.

## Materials and Methods

### Experimental Design

This prospective observational study was conducted on patients with laryngeal or hypopharyngeal cancer who received treatment at the Beijing Friendship Hospital, Capital Medical University, between September 2018 and November 2020. This study was approved by the human research Ethics Committee of Beijing Friendship Hospital, Capital Medical University (Batch number: 2018-P2-198-01). The study was conducted in accordance with the Declaration of Helsinki and Chinese Law provisions and adhered to Good Clinical Practice guidelines. According to the Response Evaluation Criteria in Solid Tumors (RECIST) criteria, patients’ clinical responses were assessed by experienced radiologists and physicians. Written informed consent was obtained from the patients and/or their authorized representatives. Inclusion criteria were as follows: primary or recurrent histologically confirmed LHSCC patients; aged above 18 years old; and fresh tissue available through either biopsy or surgical resection of the primary tumor site. Patients with cognitive impairment, mental health disorders, poor compliance, or allergic to chemotherapeutic agents were excluded. The CR cell viability and the clinical response were evaluated by technicians and clinicians double-blinded, respectively. All human tissue samples were obtained from diagnostic biopsies or therapeutic resections. Prior to surgery or biopsy, each patient signed written informed consent, allowing the excess tissue to be used for research studies.

### Tissue Processing

Upon receipt of fresh tissue, the tissue sample was into three parts for cryopreservation, fixation, and digestion for primary cell derivation. For histology, a piece was removed and immediately fixed in formalin. The fixed tissue was processed and embedded in paraffin as described previously (Driehuis et al., 2019). For primary cell culture, tissue samples were minced and incubated at 37°C in 0.125% Trypsin (Sigma, catalog no. T1426) with high glucose DMEM (Life Technologies, catalog no. 12430-054) until digested. The tissue suspension was frequently agitated and monitored for up to 60 min. The suspension was strained through a 100 μm filter, centrifuged at 300 × *g* and lysed with blood cell lysis buffer for 5 min. After washing twice with PBS, the resulting pellet was resuspended in Complete F medium and seeded in the CR culture system.

### Conditional Reprogramming Culture

Mouse embryonic fibroblast cell line 3T3-J2 (RRID:CVCL_W667; purchased from Otwo Biotech, Shenzhen, China) was cultured in complete DMEM with high glucose supplemented with 10% (v/v) FBS (Life Technologies) and 100 IU/ml penicillin, and 100 mg/ml streptomycin. In the CR system, 3T3-J2 were mitotically inactivated either by irradiation or by mitomycin C-treatment (2.5 h, 4 mg/ml final concentration, Sigma-Aldrich). Primary LHSCC cells were cultured in Complete F medium (Table 1) at 37°C in a 5% CO_2_ humidified incubator. The medium was renewed every 2 days. The cell numbers of every passage were checked by a cell counter plate.

**TABLE 1:** Formulation of complete F media.

Component	≈200 mL
Complete DMEM^1^	146 mL
F12 nutrient mixture	49 mL
Primocin	0.2 mL
EGF (10 μg/ml in PBS)	0.2 mL
Insulin (5 mg/ml in PBS)	0.2 mL
Rock inhibitor Y-27632 (10 mM in DMSO)	0.2 mL
Hydrocortisone (0.5 mg/ml in DMSO)	10 uL
Cholera toxin (8.4 μg/ml in distilled water)	0.2 mL
Adenine (2.4 mg/mL in 0.05M HCl)	2 mL
Glutamax (100×)	2 mL

*^1^Complete DMEM contains DMEM with 10% fetal bovine serum, 100 μg/ml penicillin and 100 μg/ml streptomycin.*

### Conditionally Reprogrammed Cell Derived Organoid Culture

The primary cells were collected from the CR culture system when the cells reached 70–80% confluence; the feeder cells were removed following trypsinization for 1-min. The cells at an indicated count were mixed with ice-cold Matrigel and then were loaded in the center of the well of the culture plate. After polymerization by incubating at 37°C for 30 min, a prewarmed organoid medium was added to the plate. The medium was changed every alternate day. The main organoid culture methods and composition of the organoid medium were as previously described (Driehuis et al., 2019).

### Immunofluorescence Staining

For histological examination, excised patient tissues or heterotransplanted tumors from nude mice were fixed overnight in 4% formaldehyde, dehydrated, and embedded in paraffin and followed with deparaffinization and standard hematoxylin & eosin (H&E) staining. Images were acquired on an inverted microscope (TH4-200, Olympus Optical Co., Ltd., Tokyo, Japan). Cells slides were used for indirect immunofluorescence. Briefly, cells were seeded into a 24-well plate with round cover slides (a diameter of 1 cm) in the well. After reaching 60–80% confluence, cell slides were fixed in paraformaldehyde for 15 min and acetone successively. After fixation, heat-induced antigen retrieval was performed using either citric acid solution or a microwave. The slides were then permeabilized with 0.1% Triton X-100 (Sigma) for 10 min and blocked with 1% (w/v) bovine serum albumin (BSA) for 1 h at room temperature. Following incubation overnight at 4°C with a primary antibody (anti-pan-keratin, proteintech, 26411-1-AP; anti-CD44, proteintech, 15675-1-AP), the cells were washed with PBS and incubated with secondary antibodies (Invitrogen) at room temperature for 1 h. The cells were then incubated with indicated additional stains (DAPI, life technologies D1306) for 5 min at room temperature. The samples were analyzed using a confocal microscope (LSM 880; Carl Zeiss, Germany). For immunofluorescence staining of the organoids, the whole mount staining method was performed as described previously (Hu et al., 2018). Primary antibodies used for organoids included anti-KRT5 (Santa Cruz Biotechnology; sc-32721) and anti-p63 (Abcam; ab124762). Secondary antibodies included goat anti-rabbit IgG (Alexa Fluor 594; Invitrogen; CA11012s) and goat anti-mouse IgG (Alexa Fluor 488; Invitrogen, CA11001).

### Short Tandem Repeat Amplification

Genomic DNA was extracted from cells at different passages using a DNA extraction kit (AP-MN-MS-GDNA-50; Axygen, Union City, CA, United States). A total of 21 short tandem repeat (STR) loci, including D5S818, D13S317, D7S820, D16S539, VWA, TH01, TPOX, CSF1PO, D12S391, FGA, D2S1338, D21S11, D18S51, D8S1179, D3S1358, D6S1043, PENTAE, D19S433, PENTAD, D1S1656, and Amelogenin were amplified. The fragments were amplified using PCR and separated by capillary electrophoresis using Applied Biosystems^®^ (ABI) 3730xl Genetic Analyzer (Applied Biosystems, Foster City, CA, United States), and data were automatically analyzed with the GeneMapper Software v3.2 (Applied Biosystems, Foster City, CA, United States).

### *In vitro* Drug Screening

The cells at a density of 2,000 cells/well were seeded into 96-well culture plates. After 24 h, the cells were treated with different concentrations of the drugs. Control cultures received an equal amount of DMSO (0.01 to 0.1%). 72 h after treatment. The number of viable cells was estimated by using the CCK8 assay with a slight modification. Specifically, cells treated with different concentrations of the drugs were washed twice with PBS, and then CCK8 solution (100 μL, 10 mg/mL) (Dojindo) was added into each well at 37°C for 2 h. Following incubation, the absorbance (optical density) was measured at 450 nm using a microplate reader (Thermo Scientific Multiskan FC). The values were normalized to the vehicle (100%) and baseline control (0%). For each test, if the calculated cell viability was higher than 70% or lower than 30%, an additional screen was performed for that particular drug with an adjusted dose of the drug for the cell line. *Z* factor score was used as the parameter for screen quality assessment using the following equation:


$$Z\,s\,c\,o\,r\,e=1-\frac{3\times \mathrm{SD}\,\left(\mathrm{sample}\right)+3\times \mathrm{SD}\,\left(\mathrm{negative}\,\mathrm{control}\right)}{\left|A\,v\,e\,r\,a\,g\,e\,\left(\text{sample}\right)-A\,v\,e\,r\,a\,g\,e\,\left(\mathrm{negative}\,\mathrm{control}\right)\right|}$$

Drug screens with a Z score of less than 0.3 were not used and repeated. Kill curves were generated using GraphPad^®^ PRISM version 9.0 (Graph Pad Software, Inc., La Jolla, CA, United States), and the curves were fitted using the log (Inhibitor) vs. response – Variable slope (four parameters). The half-maximal inhibitory concentration (IC50), which is an essential indicator for drug sensitivity assay, was calculated by non-linear regression of the log of concentration versus the percentage of survival, implemented in GraphPad.

### Radiation Sensitivity

The cells at a density of 2,000 cells/well were seeded in 96-well culture plates. After 24 h, cells were irradiated. The γ-ray irradiation was performed from a cobalt-60 source at a dose rate of 0.59 Gy/min at room temperature. A separate plate was used for each radiation dose. Plates were sealed air-tight and irradiated with a single fraction of 2, 4, 6, 8, and 10 Gy. After radiation, the medium was changed. Six days later, cell viability was measured using CCK8 assay. Kill curves were graphed following the method described above.

### Colony Formation Assay

The cells were seeded into six-well culture plates immediately after exposure to 0 Gy and 4 Gy of γ-ray irradiation. After 7 days of incubation, the colonies were fixed in paraformaldehyde and stained with crystal violet solution. Colonies containing more than 50 cells were counted; the relative colony-forming efficiency was calculated and plotted.

### Whole-Exome Sequencing and Bioinformatics Analysis

Whole-exome sequencing data were mapped against human reference genome GRCh37, and variants were called using the IAP pipeline^1^. To identify somatic genomic variants associated with laryngeal and hypopharyngeal cancer, WES was conducted on three paired normal/tumor CR cell lines following the protocol previously described (Li et al., 2019). We filtered out somatic single-nucleotide variations (SNVs), somatic InDels and copy number variants of tumor cells with evidence in their corresponding normal controls. The following genes were detected as cancer-associated genes and screened for all detected somatic genomic variants including, ABL1, ADAMTS12, AKT1, ALK, APC, ATM, ATR, BRAF, CASP8, CCND1, CDH1, CDH12, CDKN2A, COL1A2, COL22A1, CSF1R, CSMD3, CTNNB1, DICER1, EGFR, ERBB2, ERBB4, ESR1, EZH2, FAT1, FBXW7, FGFR1, FGFR2, FGFR3, FLT3, GNA11, GNAQ, GNAS, GRM8, HNF1A, HRAS, IDH1, IDH2, IRF6, JAK2, JAK3, KDR, KIT, KRAS, MDM2, MED1, MET, MLH1, MLL2, MPL, NAV3, NOTCH1, NOTCH2, NOTCH3, NPM1, NRAS, OR4C15, PDGFRA, PIK3CA, PKHD1L1, PRB4, PRDM9, PTEN, PTPN11, RB1, RET, RIMS2, RIPK4, SI, SLC2A13, SMAD4, SMARCB1, SMO, SRC, STK11, SYNE1, SYNE2, TP53, TP63, USH2A, and VHL.

### Heterotransplantation in Nude Mice and *in vivo* Treatment Studies

Animal experiments were approved by the Institutional Animal Care and Use Committee of Beijing Institute of Biotechnology (Ethics approval code IACUC-DWZX-2019-517, approved in May 2019). Four to 5-week-old female BALB/C-nude mice were procured from SPF (Beijing) Biotechnology Co., Ltd. Mice were cared for in accordance with the National Institute of Health (NIH) Guide for the Care and Use of Laboratory Animals. The mice were housed under specific pathogen-free conditions, at a temperature of 24°C with a relative humidity of 50–60%, under a 12-h-light/12-h-dark schedule. Animals were provided *ad libitum* access to standard rodent food and tap water. Mice were subcutaneously injected with 5 × 106 of primary cancer cells in the right flank (0.2 mL cell suspension per mouse). Six weeks after tumor cell inoculation, tumors were removed, and tumor tissues were fixed in 4% formaldehyde, embedded in paraffin, and subjected to an H&E staining procedure.

For *in vivo* treatment assay, tumor-bearing mice were established following the above-mentioned method. When tumors reached approximately 200 mm^3^, the mice were randomized into three groups (*n* ≥ 3/group) according to tumor volumes and body weights. The treatments included vehicle control, 4 mg/kg cisplatin by intraperitoneal (i.p.) injection twice a week, 5FU (100 mg/kg/week; i.p.), and paclitaxel (30 mg/kg/week; i.p.). Tumor volumes were measured using an electronic Vernier caliper and calculated with the formula *V* = π(length×width^2^)/6. On the 28th day after the first treatments, mice were weighed and then euthanized with CO_2_ asphyxia. Subsequently, the tumors were harvested, weighed, and photographed.

### Statistical Analysis

Statistical analysis was performed using GraphPad Prism 9.0 (GraphPad Software, Inc). All *in vitro* experiments were performed in triplicate and repeated three times. Data were expressed as mean ± standard deviation (SD). A two-tailed Student *t*-test was used to analyze differences between two groups. One-way analysis of variance (ANOVA) with Bonferroni correction was used to analyze multiple groups. The mean ± standard deviation was presented in all graphs, and raw data points were indicated. *P*-values of < 0.05 were considered statistically significant.

## Results

### Establishment of Patient-Derived Matched Primary Normal and Laryngeal and Hypopharyngeal Squamous Cell Carcinoma Conditional Reprogramming Cells

**FIGURE 1:**
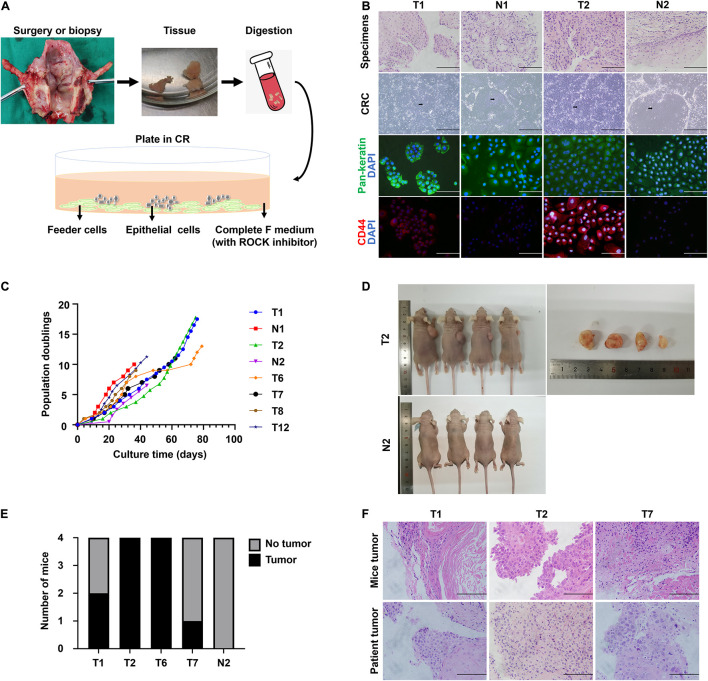
The establishment of CR system from patient-derived tissues and the characteristics of CRC lines. **(A)** Schematic representation of the digestion and initial culture condition of CRCs. Tissue obtained via biopsy or resection was collected, minced, digested using trypsin, and subsequently inoculated. **(B)** Hematoxylin and eosin (H&E) staining of primary tissue of T1, N1, T2, and N2, bright-field microscopic images of the corresponding CRCs, and immunofluorescence for pan-keratin and CD44 markers of corresponding cell slides. Black arrow, conditionally reprogrammed cells. White arrow, feeder cells. Scale bars for HE staining and immunofluorescence images, 125 μm. Scale bars for bright-field microscopy images, 500 μm. **(C)** The CRC lines were passaged repeatedly and continued to proliferate with a steady growth rate. The cell number was recorded at each passage, and a plot of population doublings versus time (days) was constructed. **(D)** Representative images of mice bearing tumors and the dissected tumors were shown. **(E)** Four independent mice were injected subcutaneously with 4 tumor CRC lines and 1 normal CRC line, and the number of mice developing tumors was depicted. **(F)** Hematoxylin and eosin (H&E) stained images of mice tumors resembled those of the corresponding patient tumors.

We obtained normal and tumor samples from patients with a histopathologically confirmed diagnosis of laryngeal or hypopharyngeal squamous cell carcinoma. The media compositions were optimized to propagate primary cells based on the published protocols for CR (Liu et al., 2017). Conditions that were successful in growing breast and prostate epithelium were refined on head and neck tissues. Briefly, the epithelial layer from the surgical specimen was microdissected to eliminate fat and muscle, digested in trypsin, and filtered. The resulting cell suspension was then seeded onto an NIH-3T3 fibroblast feeder layer in Complete F Medium (Figure 1A). The detailed composition of Complete F Medium was listed in Table 1. Within the first few days after inoculation, the adherent LHSCC cells exhibited small colonies and progressed into cell islands. As illustrated in Figure 1B, the normal conditionally reprogrammed cells (CRCs) formed tight colonies surrounded by feeder cells, similar to the previously established CR cell lines (Liu et al., 2012; Palechor-Ceron et al., 2013). CRCs were organized in a regular arrangement and exhibited a heterogeneous cell population composed of small, dark, hexagonal cells and some large and flat cells (Palechor-Ceron et al., 2019). In contrast, malignant cells displayed a unique phenotype consisting of larger individual cells with prominent intercellular junctions and a highly homogenous population of dark, hexagonal cells (Palechor-Ceron et al., 2019). Supplementary Figure 1 depicted the phase-contrast images of the six CRCs at different passages.

We received biopsy or surgical samples from 50 patients with LHSCC, intending to establish matched normal and tumor CRCs. Patient clinical characteristic data corresponding to established LHSCC CR cell lines was summarized in Supplementary Table 1. The median age of the patients was 63.7 years. Clinically, 74% (37/50) of patients exhibited T3/T4 grade tumors, according to the American Joint Committee on Cancer (AJCC, 8th edition, 2017). Additionally, 44% (22/50) of patients had lymph node metastasis. Tumor and normal CRCs were established from 56% (28/50) and 71.4% (30/42) patients, respectively (Table 2). The established criteria for CRC were long-term (>10 passages) proliferation and successful cryopreservation and recovery. These tumor CRCs were designated as T1, T2, T3, and so on, and the corresponding normal epithelium-derived CRCs were indicated by N1, N2, N3, and so on.

**TABLE 2:** Success rate of CRC culture.

Specimen	Growth (%)
Tumor tissue	28/50 (56%)
Larynx	20/32 (62.5%)
Hypopharynx	8/18 (44.4%)
Normal tissue	30/42 (71.4%)
Total	58/92 (63.0%)

The histological features were compared between CRCs and original tumors using H&E sections. CRCs exhibited similar histological patterns to their original tumors (Figure 1B). In consideration of the heterogeneity of tumor tissues, the purity of epithelial-derived cells in CRCs was evaluated using immunofluorescence. Pan-keratin was used as an epithelial marker. Positive staining of pan-keratin was found in all cells (Figure 1B). The expression of CD44, a well-known stem cell marker for head and neck cancer (Leinung et al., 2015), was higher in tumor CRCs than in its matched normal CRCs (Figure 1B).

Conditional reprogramming method conditionally induces long-term and stable proliferation. On average, CRC could form small colonies within 2–5 days and be passaged within 5–7 days. After the first passage, CRC typically proliferated at a stable rate, being passaged every 2–5 days with a split ratio of 1:2. For instance, as shown in Figure 1C, primary cells were growing at different rates, which were passaged every 2–7 days after reaching confluence in CR co-culture and continued to proliferate at a steady rate for at least 40 days with 10 population doublings till the end of the experiment. STR analysis confirmed that the primary cells were genetically stable up to a span of around 10 passages (Table 3).

**TABLE 3:** STR analysis of three representative CRCs at different passages.

Cell lines	STR alleles									AMEL
	
	D5S818	D13S317	D7S820	D16S539	VWA	TH01	D21S11	TPOX	CSF1PO	
T1 P5	10 13	9 12	8 12	9 9	16 19	9 9	29 29	8 8	9 11	X Y
T1 P15	10 13	9 12	8 12	9 9	16 19	9 9	29 29	8 8	9 11	X Y
T3 P3	11 12	8 12	9 10	9 12	16 18	6 8	28 31.2	8 11	11 11	X Y
T3 P12	11 12	8 12	9 10	9 12	16 18	6 8	28 31.2	8 11	11 11	X Y
T12 P2	13 13	9 10	8 9	9 12	18 18	7 9	30 30	11 12	11 12	X Y
T12 P12	13 13	9 10	8 9	9 12	18 18	7 9	30 30	11 12	11 12	X Y

*P, passage.*

The tumorigenic potential of the cultured LHSCC cells was evaluated by subcutaneous transplantation of the tumor CSCs into nude mice. Transplantation in all four tumor lines yielded macroscopically visible tumors after 6 weeks in at least 1 of 4 mice (*n* = 4 for each CRC line; Figures 1D,E), while injection with the normal CSCs did not result in outgrowth. H&E staining of the tumors revealed stratification and keratinization characteristics of LHSCC (Figure 1F). The tumor cells exhibited atypia, as observed in cancerous cells. Nuclear pleomorphism was also observed. Besides, muscle invasion was detected in one case. Together, LHSCC CRC lines retain tumorigenic potential and form xenografts with similar characteristics to the parental tumor.

### Conditionally Reprogrammed Cell as a Platform for Chemotherapy and Radiotherapy Sensitive Assay

**FIGURE 2:**
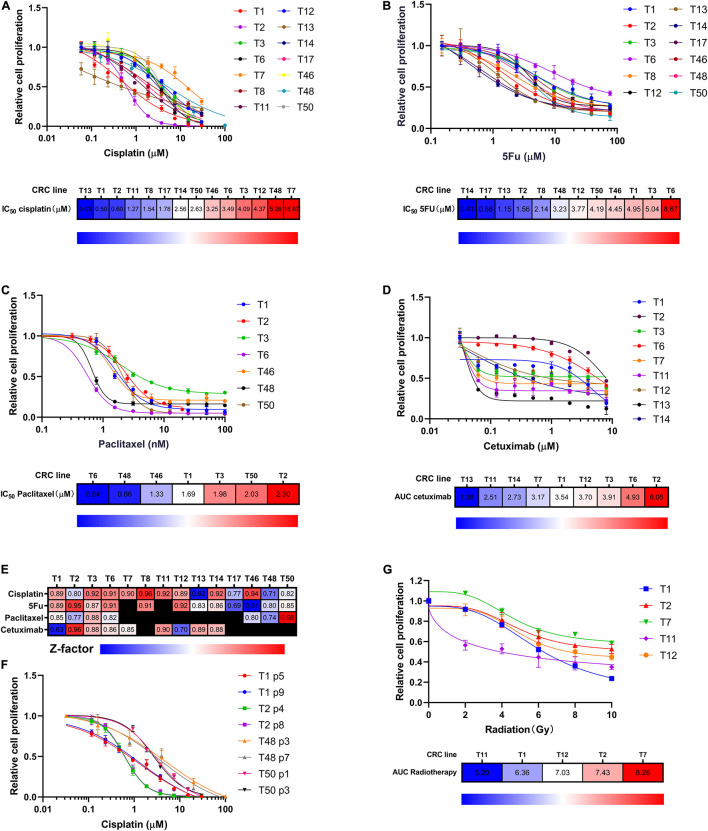
CRC lines as a platform for chemotherapy and radiotherapy sensitivity assay. **(A–C)** CRC lines revealed variable sensitivity to cisplatin, 5FU, and paclitaxel. Relative cell proliferation was plotted on the *y*-axis for different concentrations of drugs (*x*-axis). Heat maps indicated the CRCs ranking according to drug IC50. Red indicated high IC50 values; blue indicated low IC50 values. **(D)** CRC lines exhibited variable sensitivity to cetuximab. Relative cell proliferation was plotted on the *y*-axis for different concentrations of cetuximab (*x*-axis). Heat map showing the CRC lines ranked based on cetuximab sensitivity as measured by AUC. Red indicated high AUC values; blue indicated low AUC values. **(E)** Heat map showing Z factor scores of the performed drug screens for all drugs and all CRC lines presented in this study. **(F)** The consistency of cisplatin killing effects among different-passage CRC lines. 4 CRC lines were used to compare. **(G)** CRC lines revealed differential sensitivity to radiation. Relative cell proliferation was plotted on the *y*-axis for different amounts of radiation, ranging from 0 to 10 Gy (*x*-axis). Heat map showing the CRC lines ranked according to radiotherapy sensitivity as measured by AUC. Red indicated high AUC values; blue indicated low AUC values.

We exposed multiple CRC lines to cisplatin, 5FU, paclitaxel, and cetuximab, drugs currently used to treat patients with LHSCC. The *in vitro* concentration was based on the peak exposures observed at the highest clinically recommended doses delivered as a single administration (Liston and Davis, 2017). We observed differential sensitivity of the CRC lines to these compounds (Figures 2A–D). Based on the measured IC50 values, we ranked the CRC lines tested for cisplatin, 5FU, and paclitaxel (Figures 2A–C). For the sensitivity of cetuximab, the area under the curve (AUC) was applied as an alternative to IC50 values because the curvature of the kill curve was not suitable to calculate IC50 (Figure 2D). To assure the quality of the drug screening experiments, a *Z*-score, a parameter of assay quality (Zhang et al., 1999), was implemented and calculated for each drug screen (Figure 2E). The average *Z*-score was 0.84 (0.61–0.98), indicating an experimentally robust assay. To ensure the consistency of drug response between cells of different passages, 4 CRC lines in different passages were treated with cisplatin. Kill curves of the same CRC line in different passages resembled each other very well (Figure 2F).

Radiotherapy is another major treatment modality for LHSCC. Thus, we investigated the sensitivity of CRCs to ionizing radiation. Kill curves of radiotherapy were drawn, and AUC values were calculated and ranked among the five tested CRC lines (Figure 2G). Differential radiotherapy responses were observed among the CRC lines. The findings suggested that CRCs had the potential to reflect patients’ clinical responses to radiotherapy.

### Tumor Conditionally Reprogrammed Cells Recapitulate Genetic Alterations Identified in Laryngeal and Hypopharyngeal Squamous Cell Carcinoma

**FIGURE 3:**
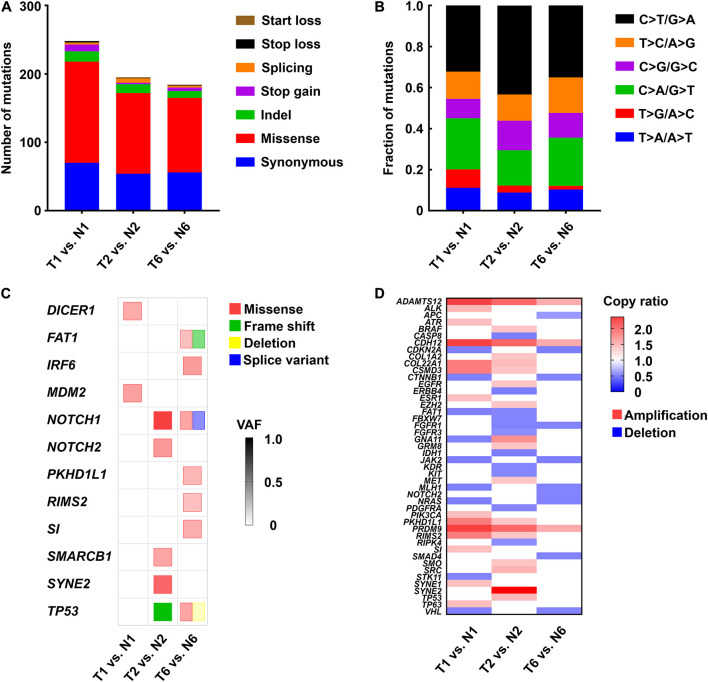
Genetic alterations identified by whole-exome sequencing in 3 paired tumor and normal CRC lines. **(A)** The number and type of somatic mutations. **(B)** The spectrum of mutations in LHSCC. **(C)** Mutations detected in LHSCC-derived CRCs that were sequenced using whole-exome sequencing. The color of the square indicated the type of mutation detected: missense (red), frameshift (green), deletion (yellow), splice variant (blue). Color intensities indicated the variant allele frequency (VAF) of the detected genetic alteration. **(D)** Heat map of copy number variant (CNV) of tumor-associated genes. Red indicated amplification with copy ratio > 1; blue indicated deletion with copy ratio < 1.

To determine whether the CRCs recapitulated genetic alterations found in LHSCC, whole-exome sequencing was conducted on matched tumor and normal CRCs from 3 patients with LHSCC. In general, the mean sequencing depth was 96.34×, and a mean of 90.99% of the target sequence was covered to a depth of at least 20× (Table 4). The somatic mutation load per subject varied significantly in LHSCC (mean 208, range 187–248; Figure 3A). The spectrum of mutations was also illustrated in Figure 3B.

**TABLE 4:** Summary of whole-exome sequencing data of six CR cell lines.

Sample	Average sequencing depth on target	Mapping rate on genome^1^ (%)	Fraction of target covered ≥ 10x (%)	Fraction of target covered ≥ 20x (%)
N1	93.08	99.84	96.45	91.28
T1	113.06	99.89	96.86	92.43
N2	92.59	99.85	96.25	90.79
T2	101.48	99.88	96.26	90.81
N6	79.18	99.8	95.76	89.27
T6	98.67	99.88	96.38	91.35
Average	96.34	99.86	96.33	90.99

*^1^Based on NCBI human reference genome GRC Build 37 (hg19).*

A mutation lists were filtered for those genes that are most commonly affected in LHSCC (Stransky et al., 2011). We scrutinized all single nucleotide variants (SNV) and small insertions or deletions (Indels) throughout the genome in the tumor and normal CRC lines. Using this criterion, we detected pathogenic mutations in 2, 5, and 7 LHSCC cancer-associated genes, in T1, T2, and T7 lines, respectively (Figure 3C). The tumor CRC lines also revealed SNVs and Indels that were absent from the normal CRC lines. The most commonly mutated gene in LHSCC, TP53, was genetically altered in 2 of the 3 tumor lines. The other tumor line, though without TP53 mutation, exhibited the mutation of MDM2, an oncogene encoding protein MDM2 binding and inhibiting P53. Thus, all the 3 tumor lines suffered from the functional alteration of P53. NOTCH1 was altered in 2 of the 3 CRC lines. Genes affected in one case include DICER1, FAT1, IRF6, MDM2, NOTCH2, PKHD1L1, RIMS2, SI, SMARCB1, and SYNE2. In the T6 line, we sequenced different mutation types in FAT1, NOTCH1, and TP53, such as missense, frameshift, deletion, or splice variant. Normal CRC lines lacked these genetic alterations, confirming they consisted of non-tumor cells. The copy number variants of cancer-associated genes were also represented in Figure 3D.

TP53 gene loci of the above CRC lines were sequenced. In detail, T1 exhibited no TP53 mutation. T2 harbored TP53 exon deletion mutation; T6 harbored TP53 exon deletion mutation and a distinct SNV, Pro72Arg, which was a defined bad mutational status indicating cisplatin-resistance (Bergamaschi et al., 2003). According to previous studies, resistance to cisplatin-based chemotherapy was positively correlated with the TP53 mutation burden (Bergamaschi et al., 2003). T1, T2, and T6 CRC lines were used to test the cisplatin treatment sensitivity. We found that CRC line T1 had the lowest IC50 than the others. Notably, T6 with two TP53 mutations propagated better than T2 with a single TP53 mutation under the same concentration of cisplatin, indicating that T6 was resistant to cisplatin treatment, and T2 was relatively sensitive to cisplatin (Figure 2A). These findings suggested that whole-exome sequencing and CRC lines derived from patient samples could predict response or resistance to individual therapy and could be used to evaluate target therapy based on gene mutation.

### Relationship of Drug Responses Between *in vitro* Conditionally Reprogrammed Cells and Xenografts in Nude Mice

**FIGURE 4:**
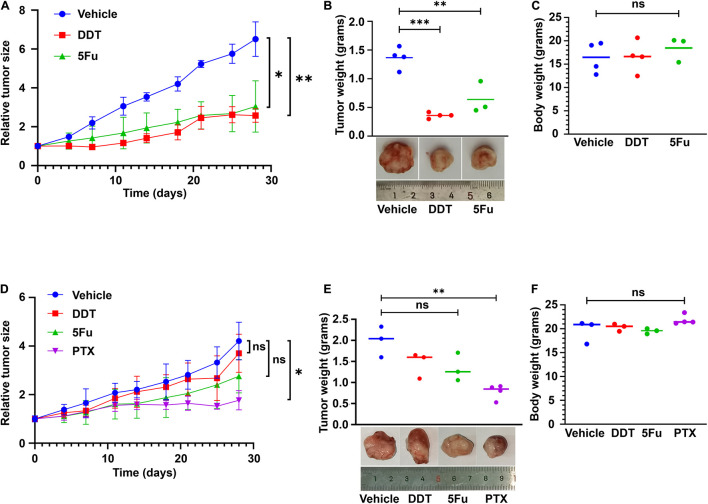
CR cell-derived xenografts used for drug testing. **(A)** T2 cell-derived xenografts were treated with vehicle control, cisplatin (DDT), and 5FU beginning from the same day after grouping for 4 weeks. Tumor sizes were measured as indicated. Mean ± SEM (*n* ≥ 3) was measured. **(B)** The tumor weight at the end of the treatment was plotted. Representative images of the tumor were presented. **(C)** Mouse body weights were also compared, with no significant difference. **(D)** T6 cell-derived xenografts were treated with vehicle control, DDT, 5FU, and paclitaxel (PTX) beginning from the same day after grouping for 4 weeks. Tumor sizes were measured as indicated. Mean ± SEM (*n* ≥ 3) was measured. **(E)** The values of tumor weight at the end of the treatment were plotted. Representative images of the tumor were presented. **(F)** Mouse body weights were also compared, with no significant difference. ^∗^*P* < 0.05, ^∗∗^*P* < 0.01, ^∗∗∗^*P* < 0.001.

Following *in vitro* findings of the routine chemotherapy agents against LHSCC, we then validated their efficacy in suppressing the growth of xenograft tumors *in vivo*. As confirmed *in vitro*, T2 was relatively sensitive to cisplatin and 5FU, while T6 was relatively resistant to the two drugs (Figures 2A,B). Hence, the same doses of cisplatin (DDT) or 5FU were administered to nude mice bearing xenografts of T2 or T6 and compared with their corresponding vehicles (Figures 4A,D). The tumor weight and mouse body weight at the endpoint were also measured (Figures 4B,C,E,F). Under the same treatment, the growth of T2 xenografts was inhibited evidently, while the growth of T6 xenografts was not hampered significantly compared with normal controls. Moreover, T6 was relatively sensitive to paclitaxel (PTX) compared to the others according to the previous *in vitro* drug response test (Figure 2C). The growth of xenografts was markedly suppressed with PTX administration, rather than DDT or 5FU (Figure 4D). Hence, the CRCs and xenografts exhibited consistent drug responses.

### Conditionally Reprogrammed Cell Derived Organoids and the Application for Drug Testing

**FIGURE 5:**
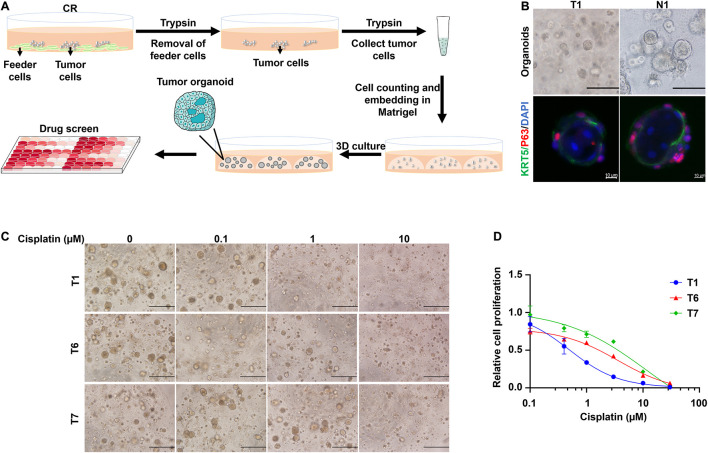
CRCs could form organoids, which could be further used for drug testing. **(A)** Schematic representation of the culture of CRC-derived organoids and further application for drug testing. **(B)** Bright-field microscopic images of CRC derived organoids (scale bar, 125 μm) and immunofluorescence analysis (scale bar, 10 μm) for the basal cell marker KRT5 (green) and p63 (red). Nuclei were counterstained with DAPI (blue). **(C)** Bright-field microscopic images of CRC derived organoids treated with different concentrations of cisplatin. Scale bar, 500 μm. **(D)** Kill curves of cisplatin to T1, T6, and T7 organoids.

Traditionally, 3D cultures have represented a widespread system to recapitulate the structural organization of primary tissues. CRCs could be transformed into organoids using the embedded method. In brief, the feeder cells were removed by first trypsinization from the CR culture system, and then the primary cells were collected by second trypsinization. Next, cells were mixed with Matrigel, and the Matrigel-cell mixture seeded into the well of the culture plate. After solidifying the gel, a dome-like structure was formed to provide the cells with a 3D growing environment. The viable cells could proliferate into spheres under this condition: CRC derived organoids (Figure 5A). Consistent with previous studies performed on head and neck cancers (Driehuis et al., 2019), CRCs successfully formed spheres that resembled the morphology of head and neck cancer organoids. The non-malignant cells expanded and re-associated to spheres of approximately 100 μm with a mass-like morphology and polarized growth (Figure 5B), while smaller spheres distinguished their malignant counterpart and a relatively slow growth rate. Immunofluorescence (IF) analysis revealed that the organoids were composed of basal cells expressing KRT5 and p63 in the outer cell layer. In contrast, keratinized and differentiated cells with enlarged nuclei were inside the organoids (Figure 5B). Furthermore, cisplatin was used to treat tumor organoid T1, T6, and T7. Bright-field images of the organoids under different drug concentrations were shown in Figure 5C. Organoids treated with 1 μM cisplatin were smaller and sparsely distributed than those treated with 0.1 μM cisplatin. Kill curves were plotted (Figure 5D). The IC50 values to cisplatin of T1, T6 and T7 were 0.50 μM, 3.6 μM and 11.6 μM, respectively. Hence, the sensitivity of the three tumor organoids to cisplatin ranked as T1 > T6 > T7. This rank was consistent with the *in vitro* CRCs responses (Figure 2A).

### Relationship Between *in vitro* Conditionally Reprogrammed Cells Responses and Clinical Responses: Special Cases

**FIGURE 6:**
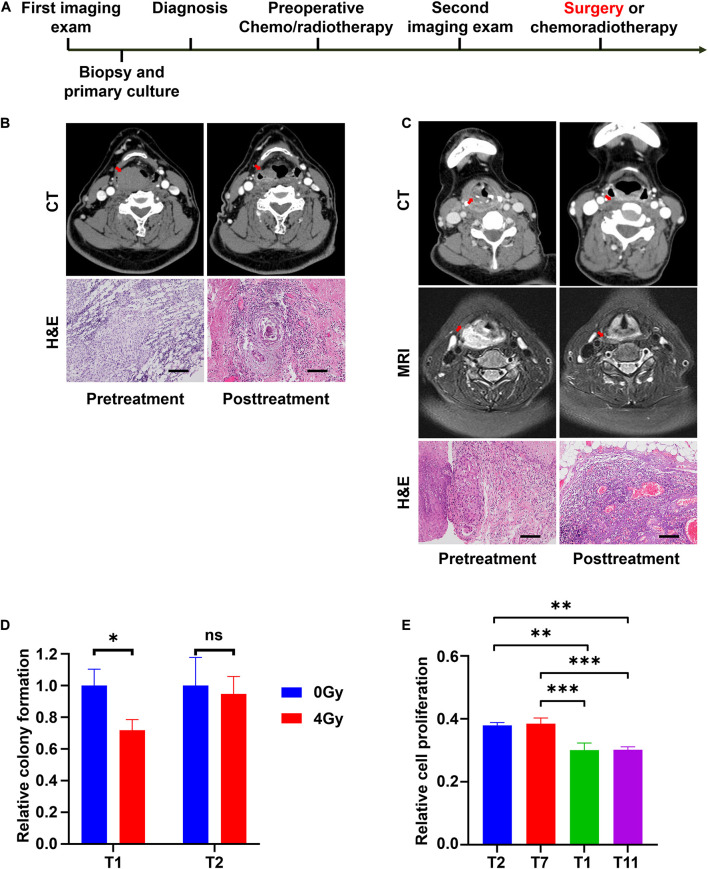
CRCs reflect the clinical outcomes of representative patients with LHSCC. **(A)** The timeline of diagnosis and treatment procedure for patients receiving preoperative chemotherapy or radiotherapy before surgery. **(B)** CT images and microscopic images of tumor tissues of patient T11 before and after chemotherapy. The size of the primary tumor, indicated by a red arrow, decreased more than 50%. Microscopic morphologies indicated pathologic complete response after chemotherapy. Scale bar, 250 μm. **(C)** CT and MRI images and microscopic images of tumor tissues of patient T13 before and after chemotherapy. The size of the primary tumor (red arrow) decreased. Microscopic morphologies indicated pathologic complete response after chemotherapy. Scale bar, 250 μm. **(D)** Relative colony formation of T1 and T2, 7 days after treatment with 0 Gy or 4 Gy radiation. ^∗^*P* < 0.05. **(E)** Relative cell proliferation of T1, T2, T7, and T11, 3 days’ culture after treatment with 4 Gy radiation. ^∗∗^*P* < 0.01, ^∗∗∗^*P* < 0.001.

To demonstrate the relationship between *in vitro* and clinical responses, four special cases were selected. Their corresponding CRC lines were derived from their biopsy tissues. After diagnosed with LHSCC, they received chemo/radiotherapy prior to surgery. Before and after the chemo/radiotherapy, imaging examinations were conducted twice to evaluate their clinical responses to the treatment according to the RECIST criterion (Eisenhauer et al., 2009). The timeline of diagnosis and treatment procedure was depicted in Figure 6A. An overview table of therapy responses of 6 representative tumors and corresponding *in vitro* models was also provided (Table 5).

**TABLE 5:** Overview of therapy responses of six representative tumors and corresponding *in vitro* models.

Sample	CR	Xenograft	Organoid	Clinical response
T1	S: DDT	NA	S: DDT	NA
T2	S: DDT and 5FU R: RT	S: DDT and 5FU	NA	R: RT
T6	S: PTX R: DDT and 5FU	S: PTX R: DDT and 5FU	R: DDT	NA
T7	R: DDT and RT	NA	R: DDT	R: RT
T11	S: DDT and RT	NA	NA	S: DDT and RT
T13	S: DDT	NA	NA	S: DDT and RT

*S, sensitivity; R, resistance; DDT, cisplatin; RT, radiotherapy; PTX, paclitaxel; NA, not applicable.*

The patient from whom T11 was derived had hypopharyngeal carcinoma (stage T3N0M0). The patient was treated with chemoradiotherapy (two sessions of cisplatin and radiotherapy dose of 40 Gy) prior to surgery because of the strong personal willingness of the laryngeal preservation approach. The tumor partially responded to the treatment as assessed from imaging examination (Figure 6B), and a laryngeal preservation surgery was conducted later. Fortunately, a pathologic complete response (pCR) was achieved after postoperative pathologic examination of the resected tissue (Figure 6B). This indicated that T11 was sensitive to the treatment of cisplatin and radiotherapy *in vivo*. Indeed, CRC line T11 was relatively sensitive to cisplatin from our *in vitro* drug screening assay. Similarly, patient T13, who exhibited the highest sensitivity to cisplatin, was diagnosed with hypopharyngeal carcinoma (stage T4N1M0) and was also treated with 2 sessions of cisplatin and a radiotherapy dose of 40 Gy prior to surgery. After the removal of the residual primary tumor, the pathologic examination demonstrated a pCR (Figure 6C).

The patient of T2 presented with laryngeal squamous cell carcinoma (stage T4N2M0) was treated with preoperative radiotherapy. However, the patient showed progressive disease status shortly after radiotherapy of 40 Gy, and a total laryngectomy was performed. Postoperative radiotherapy of a dose of 26 Gy was administered because of lymph node metastasis. Unfortunately, the patient succumbed to locoregional recurrence 4 months later. Patient T7 was diagnosed with hypopharyngeal carcinoma (stage T4N2M0). Progressive disease status was observed after preoperative radiotherapy of 40 Gy, hence partial hypopharyngectomy and total laryngectomy were performed on the patient. Postoperative radiotherapy of a dose of 26 Gy was given because of positive margins. The patient was last followed up 1 year after the end of treatment. There were no signs of recurrence to this point. Further, follow-up would be conducted to observe any remission. The *in vitro* sensitivity to radiation, colony formation assay, and cell proliferation assay were performed on the CRCs. After exposing to 4 Gy radiation, T1 exhibited a significant inhibition in colony formation efficiency, while T2 did not (Figure 6D). Besides, CRCs T1, T2, T7, and T11 were treated with 4 Gy radiation. After 3 days’ culture, their relative cell proliferation was detected. The result indicated that relative cell proliferation of T2 and T7 was significantly higher than that of T1 and T11 after radiation (Figure 6E). Based on these findings, T2 and T7 presented resistant to radiotherapy. Consistently, the clinical history showed concordance with the *in vitro* findings.

## Discussion

Management of LHSCC is highly complicated and mandates multidisciplinary care (Ringash, 2015). Precision oncology aims to identify and target tumor-specific aberrations with effective therapeutic strategies for individual cancer patients. Currently, in precision oncology, the recommendation of molecularly targeted drugs is primarily based on the genomic profile of a drug-target gene as a therapeutic indicator (Bailey et al., 2018). The drive toward precision oncology has significantly increased attention in adapting *in vitro* tumor models for patient-specific therapies, drug response assessment, and clinical management. Various *in vitro* models for LHSCC were developed to guide patient-specific therapies in the past decades, including patient-derived primary tumor cells, 3D culture spheres, patient-derived xenotransplantation, and tumor organoids (Seshadri et al., 2009; Dohmen et al., 2015; Méry et al., 2017; Karamboulas et al., 2018; Tanaka et al., 2018). Although promising, those models had numerous limitations for further clinical practice (Dohmen et al., 2015). Success rate, time window, accuracy, and cost-effectiveness are considered essential criteria to the establishment of *in vitro* tumor models. Herein, using CR technology, we established patient-derived LHSCC cells and investigated the clinical implications of them.

In this study, the primary cells were successfully established from 56% tumor samples and 71.4% normal epithelial samples under CR system conditions. We confirmed that the primary cells were epithelial-derived cells expressing pan-keratin, and tumor cells exhibited stemness expressing tumor marker CD44. STR results of the CRCs differed from each other and did not match any cell lines in the database of DSMZ (Deutsche Sammlung von Mikroorganismen und Zellkulturen)^2^, German Collection of Microorganisms and Cell Cultures GmbH. STR results of each cell lines remained stable at different passages. Through tissue dissociation and cells’ amplification in the CR culture system, we were able to obtain 10^6^ cells from each individual endoscopic biopsy or tumor sample within 2 weeks. The cells could be cultured for more than 40 days and 10 passages, with a stable proliferation rate during the extended period. Moreover, the proliferation of cells was not affected by repeated cycles of cryopreservation and resuscitation. Collectively, the CR system contented the criteria for continuous cell culture from LHSCC tumor samples. Besides, sufficient cell numbers could be acquired for further assays such as drug screening, sequencing, or xenografts.

In the present study, the drug and radiation sensitivity of some established CRC lines were tested. Four mostly used drugs in treating LHSCC patients were used in the drug screening. Various drug response was observed. We ranked the CRC lines by their IC50 values for each drug. The CRC lines with higher IC50 values were considered to be more resistant to the specific drug than those with lower IC50 values. There was no obviously regular pattern of the sensitivity to each drug among the CRC lines. These findings motivated to explore the clinical responses using CR techniques. These results were robust due to reliable Z-scores and biological duplication, and they were applied further in the comparison to the drug responses of other *in vitro* models.

As a milestone for the CR technique, Liu et al., successfully generated continuous cell cultures from tumor samples in a patient with recurrent respiratory papillomatosis and identified Vorinostat as a therapeutic agent using *in vitro* chemosensitivity testing (Yuan et al., 2012). CR technique played a crucial role in guiding clinical administration, though only 1 case was involved. Most of the patients included in this study received surgical resection of the tumor as primary treatment, making it impossible to assess their chemoradiotherapy response outcomes by RECIST criterion (Eisenhauer et al., 2009). Only 4 cases treated with preoperative chemo/radiotherapy were selected to validate the relationship between *in vitro* and clinical responses. And closely matched responses were observed. However, more cases are required to validate this relationship robustly. These results implied that CR cultured LHSCC could predict patients’ clinical responses to chemotherapy or radiotherapy and might serve as an excellent preclinical model for precision oncology.

Based on the whole-exome sequencing of the three pairs of CRC lines, resistance to cisplatin-based chemotherapy was also positively correlated with TP53 mutation burden, consistent with a previous study (Bergamaschi et al., 2003). Epidermal growth factor receptor (EGFR) was overexpressed in 50%∼90% of the tumors (Bossi et al., 2016), and about 15% of patients carry gene amplification of EGFR (Network, 2015). None of the sequenced cells carried EGFR mutation in this study, and only one of them (T2) had EGFR gene amplification. Cetuximab, a monoclonal antibody targeting EGFR, was used in drug screening. However, the cetuximab sensitivity of the CRC lines could not be reflected by their EGFR expression levels. Although conflicting, the result agreed with previous studies (Bossi et al., 2016).

After being heterotransplanted subcutaneously in nude mice, the primary tumor CRC lines could form tumors successfully, while the normal paired ones could not. This demonstrated the malignant potential of the tumor cells. These CR cell-derived xenografts retained the primary tumors’ histopathologic characteristics and could be used for personalized treatment just as patient-derived xenografts (PDX) did (Karamboulas et al., 2018). Moreover, CRC lines could be expanded, cryopreserved, and resuscitated *in vitro* and are repeatedly used for xenografts, which made it much more flexible and convenient than the PDX model.

Patient-derived organoids (PDOs), recapitulating the primary tissues’ genetic and molecular characteristics, have been applied to conduct high-throughput drug screening and predict the treatment responses of HNSCC (Driehuis et al., 2019). We could not ignore its disadvantage as aiming at potential clinical application. Firstly, the success rate of LHSCC organoids culture was relatively low, according to the previously published literature (Driehuis et al., 2019). We also tried to culture tumor organoids following previously described methods, but a dissatisfying success rate of 12.5% (2/16) was achieved. Secondly, a clinically significant time window was not available as required for personalized treatment decision-making, which is generally less than 2–3 weeks for preoperative and postoperative chemotherapy. It is not sufficient time for tumor organoids to proliferate to optimal cell number for drug testing. Thirdly, the organoid culture system relied on multiple expensive growth factors in the culture medium and extracellular matrix substitutes. The high expense hampered its extensive application for a less supported institute. Finally, organoid associated processes, unlike classical monolayer cell culture techniques, were special and too complicated to get started. Furthermore, to demonstrate the stemness of CRC lines, we embedded the dissociated CR primary cells in Matrigel and cultured them under the media of organoid culture (Driehuis et al., 2019). Sphere-shaped organoids could be cultured from the primary cells. A polarized growth was observed as the basal cells stratified the outer layer of the organoids while the differentiated and keratinized cells located inside the organoids. Furthermore, these CR derived organoids could also be used to test drug sensitivity.

The success rate of culturing LHSCC cells using CR system was not among the highest (Dohmen et al., 2015), though superior to PDX and organoid. This was partly due to the nature of LHSCC as follows. Most patients were aged ones with poor cell activity. Too many necrotic or fibrotic tissues which were unable to proliferate. And the primary tumor was exposed to numerous microbes that increased the probability of contamination.

In summary, we established LHSCC primary cell lines using the CR technique. The CR cell lines had retained histological and molecular characteristics and heterogeneity of the parental LHSCC. CR cell lines could be transformed into xenografts and organoids, serving as versatile *in vitro* models. Collectively, patient-derived cell model system using CR technology could be promisingly utilized in clinical decision-making and help identify personalized therapies for LHSCC.

## Data Availability Statement

The datasets presented in this study can be found in online repositories. The names of the repository/repositories and accession number(s) can be found below: https://www.ncbi.nlm.nih.gov/sra/?term=PRJNA758159.

## Ethics Statement

The studies involving human participants were reviewed and approved by the human research Ethics Committee of Beijing Friendship Hospital, Capital Medical University. The patients/participants provided their written informed consent to participate in this study. The animal study was reviewed and approved by Institutional Animal Care and Use Committee of Beijing Institute of Biotechnology.

## Author Contributions

SL and LL designed the study and experiments. YD analyzed the data. YD, JW, WJ, and MZ performed molecular and cell biological experiments. YD, JW, and MZ performed animal studies. YD and WJ collected the patients’ clinical data. YD, WJ, and LL analyzed the clinical manifestations and responses. YD, JW, and SL drafted the manuscript. All authors commented on the manuscript.

## Conflict of Interest

The authors declare that the research was conducted in the absence of any commercial or financial relationships that could be construed as a potential conflict of interest.

## Publisher’s Note

All claims expressed in this article are solely those of the authors and do not necessarily represent those of their affiliated organizations, or those of the publisher, the editors and the reviewers. Any product that may be evaluated in this article, or claim that may be made by its manufacturer, is not guaranteed or endorsed by the publisher.

## Footnotes

1
https://github.com/UMCUGenetics/IAP

2
www.dsmz.de
