# Supplementary material for: Preclinical Application of Conditional Reprogramming Culture System for Laryngeal and Hypopharyngeal Carcinoma
Source: Front Cell Dev Biol. 2021 Oct 29;9:744969. doi: 10.3389/fcell.2021.744969 (PMC8585768; doi:10.3389/fcell.2021.744969)
Supplement: Supplementary file 1 [file Data_Sheet_1.docx]

Supplementary Material

# Supplementary Figures and Tables

## Supplementary Figures


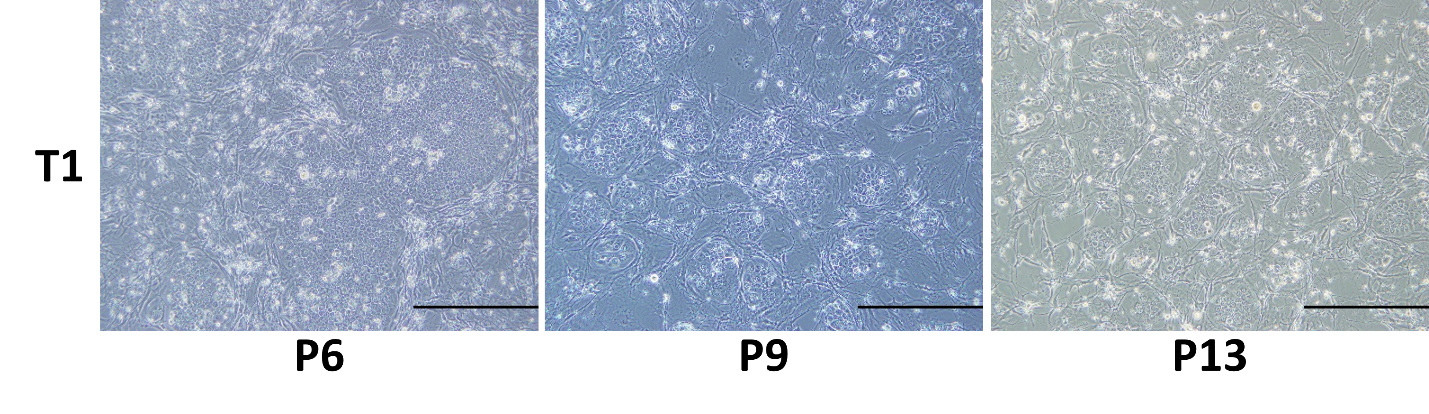


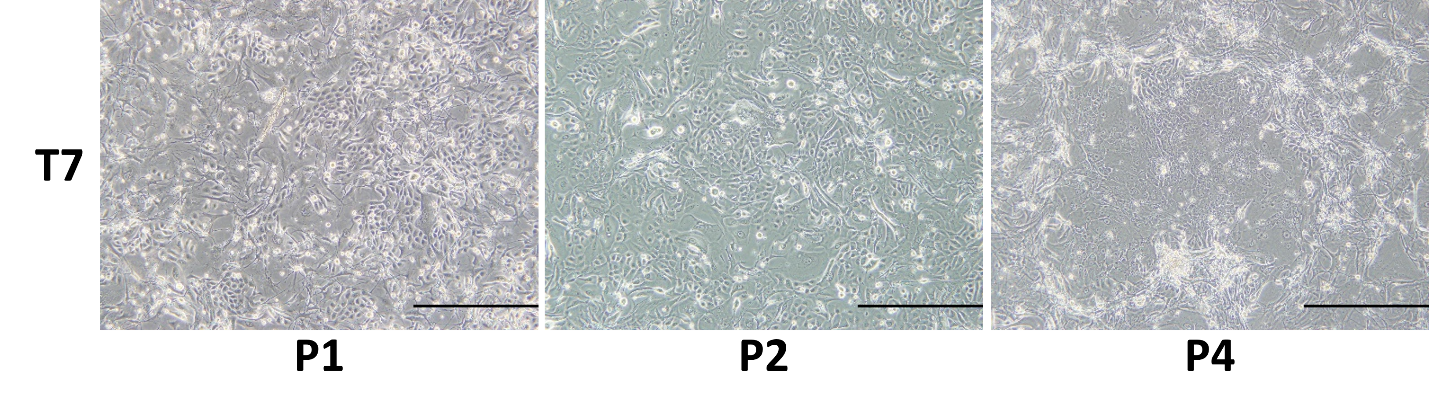

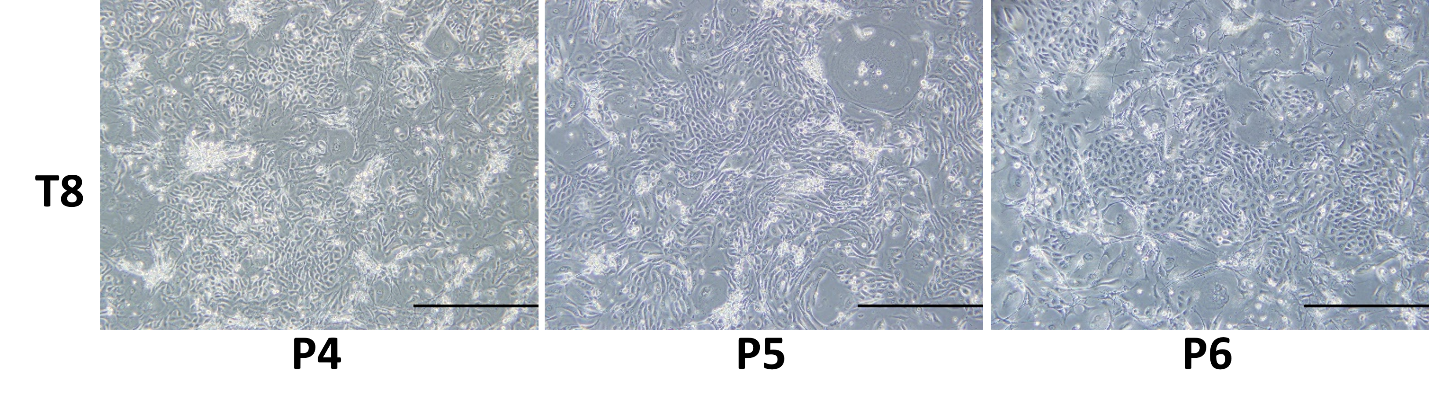

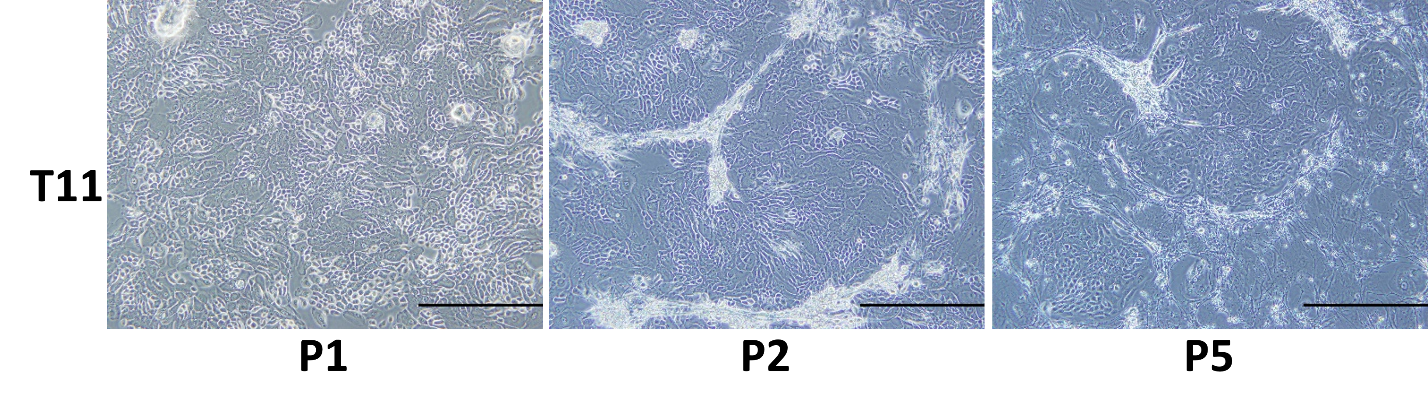

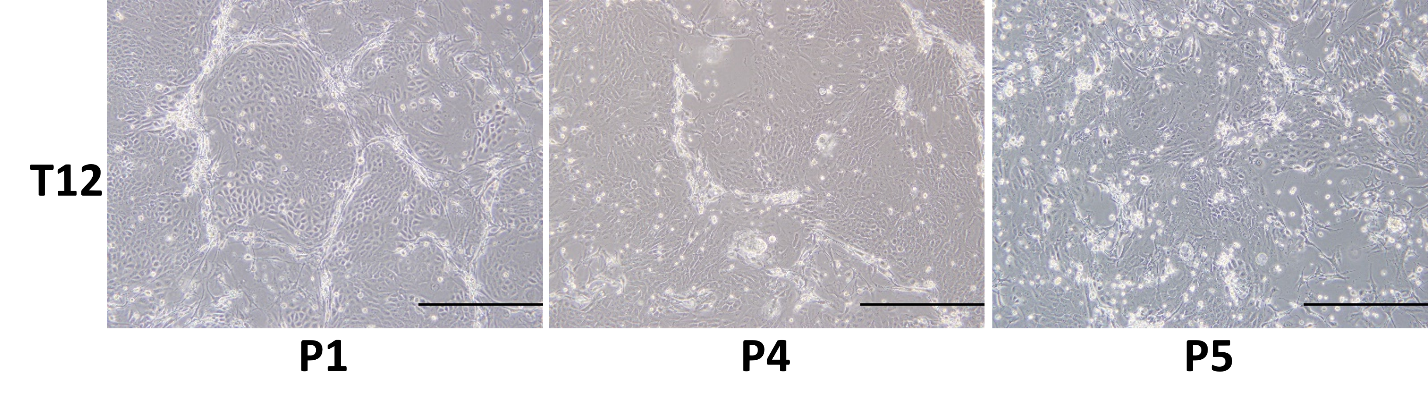

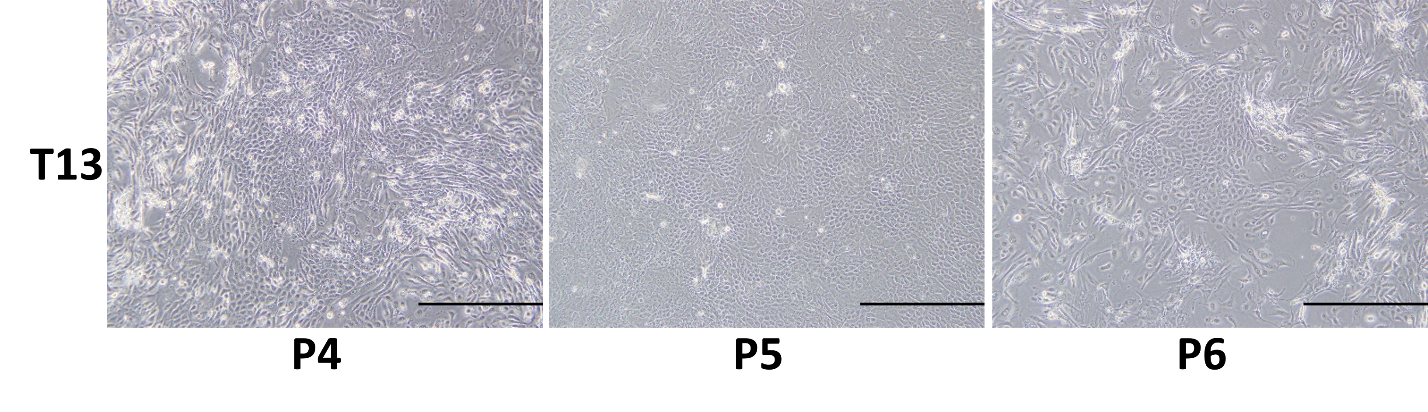


**Supplementary Figure S1.** Representative phase contrast images of CR cells.

Supplementary Table S1. Patient clinical data

| No. | gender | age | tumor location | T-stage | N-stage | pretreatment | surgery or biopsy | N/T^3^ | successful CR culture |
| --- | --- | --- | --- | --- | --- | --- | --- | --- | --- |
| 1 | male | 62 | larynx | 3 | 1 | no | surgery | N/T | N/T |
| 2 | male | 56 | larynx | 4 | 2 | RT^1^ | biopsy and surgery | N/T | N/T |
| 3 | male | 66 | larynx | 2 | 0 | no | surgery | N/T | T |
| 4 | male | 56 | larynx | 3 | 1 | no | surgery | N/T | N/T |
| 5 | male | 57 | hypopharynx | 4 | 0 | no | surgery | N/T | N/T |
| 6 | male | 49 | larynx | 2 | 0 | no | surgery | N/T | N/T |
| 7 | male | 83 | hypopharynx | 4 | 2 | RT | biopsy and surgery | N/T | N/T |
| 8 | male | 67 | larynx | 3 | 0 | no | surgery | N/T | T |
| 9 | male | 62 | larynx | 4 | 2 | no | surgery | N/T | N/T |
| 10 | male | 66 | hypopharynx | 4 | 0 | no | surgery | N/T | N/T |
| 11 | male | 69 | hypopharynx | 3 | 0 | CRT^2^ | biopsy | T | T |
| 12 | male | 67 | larynx | 4 | 2 | no | biopsy | T | T |
| 13 | male | 67 | larynx | 3 | 0 | CRT | biopsy | T | T |
| 14 | male | 61 | hypopharynx | 2 | 2 | no | surgery | N/T | T |
| 15 | male | 69 | larynx | 2 | 0 | no | surgery | N/T |  |
| 16 | male | 59 | hypopharynx | 4 | 2 | no | surgery | N/T | N |
| 17 | male | 63 | larynx | 3 | 0 | no | surgery | N/T | N/T |
| 18 | male | 64 | larynx | 3 | 0 | no | surgery | N/T | N |
| 19 | male | 67 | larynx | 3 | 0 | no | surgery | N/T | N/T |
| 20 | male | 78 | larynx | 3 | 0 | no | surgery | N/T | N/T |
| 21 | male | 69 | hypopharynx | 3 | 3 | no | surgery | N/T | N/T |
| 22 | male | 50 | larynx | 3 | 2 | no | surgery | N/T | N |
| 23 | male | 63 | hypopharynx | 3 | 0 | no | surgery | N/T |  |
| 24 | male | 76 | larynx | 3 | 0 | no | surgery | N/T | N/T |
| 25 | male | 79 | hypopharynx | 4 | 2 | no | surgery | N/T | N |
| 26 | male | 56 | larynx | 2 | 0 | no | surgery | N/T |  |
| 27 | male | 59 | larynx | 3 | 2 | no | surgery | N/T | N/T |
| 28 | male | 54 | larynx | 2 | 0 | no | surgery | N/T | N/T |
| 29 | male | 64 | larynx | 3 | 0 | no | surgery | N/T | N/T |
| 30 | male | 59 | hypopharynx | 3 | 0 | no | surgery | N/T | N |
| 31 | male | 65 | hypopharynx | 2 | 2 | no | surgery | N/T |  |
| 32 | male | 65 | larynx | 2 | 0 | no | surgery | N/T | N |
| 33 | male | 64 | hypopharynx | 3 | 2 | no | surgery | N/T |  |
| 34 | male | 63 | larynx | 3 | 0 | no | surgery | N/T |  |
| 35 | male | 69 | larynx | 3 | 0 | no | biopsy | T | T |
| 36 | male | 59 | larynx | 3 | 1 | no | biopsy | T |  |
| 37 | male | 65 | hypopharynx | 3 | 3 | CRT | biopsy | T |  |
| 38 | male | 75 | larynx | 4 | 2 | no | biopsy | T |  |
| 39 | male | 56 | hypopharynx | 2 | 2 | CRT | biopsy | T |  |
| 40 | male | 58 | hypopharynx | 4 | 1 | no | surgery | N/T |  |
| 41 | male | 65 | hypopharynx | 2 | 0 | no | surgery | N/T | N/T |
| 42 | male | 64 | larynx | 3 | 0 | no | surgery | N/T | N/T |
| 43 | male | 63 | larynx | 3 | 0 | no | surgery | N/T | N |
| 44 | male | 66 | larynx | 3 | 0 | no | surgery | N/T |  |
| 45 | male | 63 | larynx | 2 | 0 | no | surgery | N/T | N |
| 46 | male | 58 | larynx | 3 | 0 | no | surgery | N/T | N/T |
| 47 | male | 61 | hypopharynx | 2 | 1 | no | surgery | N/T |  |
| 48 | male | 63 | larynx | 3 | 2 | no | surgery | N/T | N/T |
| 49 | male | 68 | larynx | 4 | 0 | no | surgery | N/T | N |
| 50 | male | 56 | hypopharynx | 2 | 3 | no | surgery | N/T | N/T |

1. RT: radiotherapy. 2. CRT: chemoradiotherapy. 3. N/T: normal / tumor tissue
